# From lipotoxicity to pan-lipotoxicity

**DOI:** 10.1038/s41421-025-00787-z

**Published:** 2025-03-18

**Authors:** Yiping Cheng, Shanshan Shao, Zhen Wang, Qingbo Guan, Huaxue Li, Guodong Liu, Haiqing Zhang, Xiude Fan, Jiajun Zhao

**Affiliations:** 1 Key Laboratory of Endocrine Glucose & Lipids Metabolism and Brain Aging, Ministry of Education; Department of Endocrinology, Shandong Provincial Hospital Affiliated to Shandong First Medical University, Jinan, Shandong China; 2 Shandong Key Laboratory of Endocrine Metabolism and Aging, Jinan, Shandong China; 3 Shandong Institute of Endocrine and Metabolic Diseases, Jinan, Shandong China; 4 Shandong Engineering Laboratory of Prevention and Control for Endocrine and Metabolic Diseases, Jinan, Shandong China; 5 Department of Endocrinology, Shandong Provincial Hospital, Shandong University, Jinan, Shandong China

In 1994, John Denis McGarry introduced the concept of “lipotoxicity”, defining it as the cellular damage and dysfunction of tissues and organs caused by the ectopic accumulation of triglycerides and free fatty acids in non-adipose tissues^1^. At the time, due to the limitations of detection techniques and conceptual understanding, the concept of “lipotoxicity” originally encompassed only triglycerides and free fatty acids, was confined to ectopic lipid accumulation as its primary manifestation, and was considered to exert toxic effects solely through localized pathological changes in specific tissues and organs. This narrow perspective restricted the understanding of the pathophysiological mechanisms, preventive strategies, and therapeutic approaches for lipid metabolism dysfunction-associated disorders. To address these limitations, we propose the concept of “pan-lipotoxicity”, which encompasses systemic cellular damage and tissue and organ dysfunction caused by lipid accumulation or imbalances in subcomponent proportions, lipoprotein metabolism disorders, and abnormal fat distribution or function. This new term will promote a paradigm shift in etiology research, from focusing on “single lipid components” to a “lipid metabolism panorama”, and in diagnostic and treatment models, from “single disease management” to “comorbid collaborative prevention and treatment”. This shift will lay the foundation for mechanistic studies, biomarker identification, and the diagnosis and treatment of lipid metabolic dysfunction-associated diseases.

## Why the traditional concept of “lipotoxicity” needs innovation?

The traditional concept of “lipotoxicity” primarily emphasizes the detrimental effects associated with the accumulation of triglycerides and free fatty acids in tissues such as the liver, pancreas, and muscle. However, advancements in lipid metabolism research, particularly its association with various diseases, have highlighted the necessity to broaden the scope of lipid species implicated in lipotoxicity. It has been established that an excess of cholesterol, phospholipids, sphingolipids, and glycolipids significantly contributes to disease pathogenesis^2–4^. For example, elevated ceramide levels have been linked to an increased risk of multiple metabolic disorders, including diabetes, cardiomyopathy, insulin resistance, atherosclerosis, and steatohepatitis^5^. Consequently, focusing solely on triglycerides and free fatty acids fails to comprehensively address the toxic effects of various lipid molecules.

There is a pressing need for further investigation into the manifestations and mechanisms of lipotoxicity, which encompasses not only lipid accumulation but also imbalances in the proportions of lipid subcomponents, disorders of lipoprotein metabolism, and variations in fat distribution (such as central obesity and lean metabolic dysfunction-associated steatotic liver disease (MASLD)). Dysfunctions in fat tissue such as abnormal secretion of adipokines or metabolites are also critical pathogenic mechanisms for multi-organ damage and dysfunction^6–8^. Moreover, lipotoxicity is not limited to directly inducing localized pathological changes in specific organs; these local changes can also exert systemic pathological effects on distant tissues or organs through signaling pathways. For example, MASLD may influence the brain via systemic inflammation and oxidative stress, increasing the risk of cognitive impairment. This highlights the need to understand hepatic changes from a holistic perspective, considering their impact on other organs and overall metabolism. Therefore, researchers need to transcend the traditional boundaries of lipotoxicity and systematically consider the lipid metabolism panorama, analyzing intercellular and inter-organ communications, and focusing on the interactive mechanisms between metabolic pathways. This will provide valuable insights into fully understanding the role of lipotoxicity in metabolic diseases and its underlying biological mechanisms.

The concept of “lipotoxicity” is not suitable for the prevention and diagnosis of lipid metabolic dysfunction-associated diseases. For example, plasma triglyceride levels do not adequately indicate the risk of atherosclerotic cardiovascular disease (ASCVD). In addition to triglycerides and low-density lipoprotein cholesterol (LDL-C), other lipid biomarkers such as apolipoprotein B (ApoB), remnant lipoproteins, small dense low-density lipoprotein, and lipoprotein(a), have been identified to be significantly associated with ASCVD risk^9^. Relying only on routine lipid tests, such as triglycerides and LDL-C, can lead to underdiagnosis of high-risk cardiovascular events, which prevents early identification and effective preventive measures. Cardiology has long emphasized the central role of dyslipidemia, particularly LDL-C, in the prevention and management of cardiovascular diseases. However, in other critical disease areas, such as cancer and metabolic disorders, the significance of lipid metabolism abnormalities has not received equivalent attention. A more comprehensive understanding of the broad impact of lipid metabolism abnormalities across these conditions is essential. Consequently, it is crucial to move beyond the traditional “lipotoxicity”, integrating current multi-biomarker approaches and employing advanced technologies and methodologies like nuclear magnetic resonance spectroscopy and liquid chromatography-tandem mass spectrometry. These innovations facilitate the identification of novel “pan-lipotoxicity” biomarkers associated with lipid metabolic dysfunction-associated diseases, thereby optimizing screening and diagnostic frameworks.

In terms of treatment, the PROMINENT study, published in the *New England Journal of Medicine* in 2022, demonstrated that treatment with pemafibrate, a fibrate drug, did not confer cardiovascular benefits, leading to premature termination of the study. This outcome was speculated to be due to its concurrent elevation of LDL-C (12.3%) and ApoB (4.8%)^10^. Furthermore, drug development for metabolic dysfunction-associated steatohepatitis (MASH) is rapidly advancing. Research suggests that medications initially developed for type 2 diabetes may also be effective for MASH. For instance, GLP-1R/GIPR dual agonists have shown potential benefits, such as increasing energy expenditure and reducing hepatic fat content. This underscores the need to move beyond the traditional concept of lipotoxicity in guiding treatment strategies for lipid metabolic dysfunction-associated diseases. Instead, therapeutic approaches should be based on a comprehensive understanding of lipid metabolism dysregulation, allowing for the identification of novel intervention targets and the optimization of treatment strategies. Such an approach will facilitate early, precise interventions and the proactive prevention and management of lipid metabolism disorders.

**Fig. 1:**
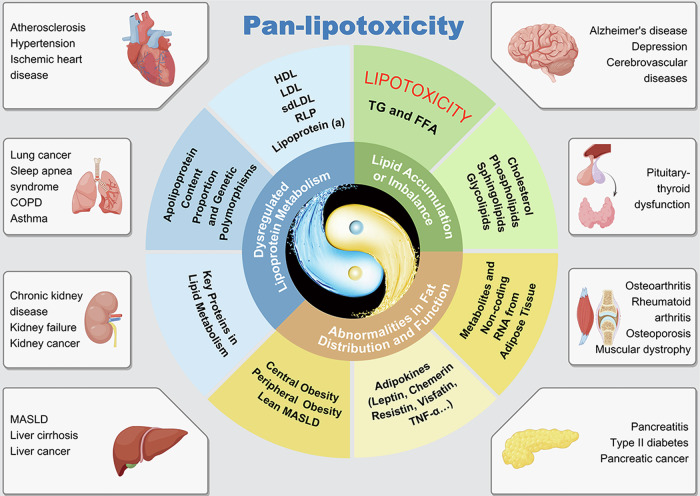
The connotation of pan-lipotoxicity. The conditions shown are representative examples; associations between pan-lipotoxicity and other diseases are not limited to those depicted. TG triglyceride, FFA free fatty acid, HDL high-density lipoprotein, LDL low-density lipoprotein, sdLDL small dense low-density lipoprotein, RLP remnant-like lipoprotein, TNF-a tumor necrosis factor-alpha, RNA ribonucleic acid, COPD chronic obstructive pulmonary disease, MASLD metabolic dysfunction-associated steatotic liver disease.

Furthermore, lipotoxicity shares a common pathological foundation, interconnected metabolic pathways, and similar pathogenic effects across different cell types, tissues, and organs. However, various disciplines tend to focus on localized lesions in isolated organs, which results in a failure to apply a systematic and holistic approach to the understanding, research, and treatment of lipid metabolic dysfunction-associated diseases. The introduction of the concept of “pan-lipotoxicity” from a systematic perspective offers a new viewpoint, urging us to explore the common core pathogenic mechanisms of lipotoxicity and achieve coordinated prevention and treatment of multiple diseases (Fig. 1).

## Scientific value and clinical significance of “pan-lipotoxicity”

The concept of “pan-lipotoxicity” broadens the scientific connotation of traditional lipotoxicity. It not only focuses on the toxic effects of free fatty acids and triglycerides, but also includes the impacts of other lipid species including phospholipids, sphingolipids, and cholesterol esters, imbalances in their subcomponent proportions, lipoprotein metabolism disorders, and abnormal fat distribution and function on overall metabolic health, rather than just a single organ. This comprehensive approach enables researchers to investigate the interactions between adipose tissue function, lipid metabolism abnormalities, and multi-organ damage from a lipid metabolism panorama, breaking down disciplinary barriers and building a panoramic, multidisciplinary lipid metabolism health management system.

Guided by the concept of “pan-lipotoxicity”, future research and clinical applications can focus on the following areas:

1. Investigating the interactions at the intercellular, inter-organ, and inter-system levels, such as interactions of cell membrane-endoplasmic reticulum-mitochondria, heart-liver-kidney, and the digestive system-cardiovascular system-central nervous system. This will elucidate the mechanisms linking lipid metabolism abnormalities with conditions like cancer and aging, providing important insights into identifying novel biomarkers and finding precise intervention targets.
2. Integrating lipidomic analysis, lipoprotein subtypes, lipid metabolism enzyme activity assays, adipokine detection, and imaging characterization of fat distribution to create a body lipid health index. This index will facilitate the systematic quantification of an individual’s lipid health status and optimize early screening and classification systems for lipid metabolism abnormalities.
3. Gathering data on lipid metabolites, lipoproteins, fat distribution, adipokines, and multi-organ function to construct a pan-lipotoxicity disease database. This database will support clinical research, and provide evidence for risk prediction, early diagnosis, and personalized interventions in pan-lipotoxicity diseases.
4. From the perspective of “differential treatment for the same disease” and “unified regulation for different diseases”, we aim to develop personalized management plans for nutrition, exercise interventions, and novel lipid-lowering drugs based on lipid metabolism abnormality profiling. Furthermore, we seek to integrate lipid metabolism with other metabolic pathways, such as glucose, purine, and energy metabolism, to develop innovative drugs targeting coordination of lipid metabolism. This approach will enable early screening, dynamic monitoring, and comprehensive management of pan-lipotoxicity diseases.

## References

[1] Lee, Y. et al. *Proc. Natl. Acad. Sci. USA* **91**, 10878–10882 (1994). 7971976 10.1073/pnas.91.23.10878 PMC45129

[2] Song, Y. et al. *Cell Metab.* **33**, 1911–1925 (2021). 34562355 10.1016/j.cmet.2021.09.001

[3] Sasset, L. et al. *Trends Endocrinol. Metab.* **27**, 807–819 (2016). 27562337 10.1016/j.tem.2016.07.005 PMC5075255

[4] Tsimikas, S. et al. *Nat. Rev. Cardiol.* **21**, 170–191 (2024). 37848630 10.1038/s41569-023-00937-4

[5] Chaurasia, B. et al. *Trends Endocrinol. Metab.* **26**, 538–550 (2015). 26412155 10.1016/j.tem.2015.07.006

[6] Petersen, K. F. et al. *N. Engl. J. Med.* **362**, 1082–1089 (2010). 20335584 10.1056/NEJMoa0907295 PMC2976042

[7] Ye, Q. et al. *Lancet Gastroenterol. Hepatol.* **5**, 739–752 (2020). 32413340 10.1016/S2468-1253(20)30077-7

[8] Scheja, L. et al. *Nat. Rev. Endocrinol.* **15**, 507–524 (2019). 31296970 10.1038/s41574-019-0230-6

[9] Duran, E. K. et al. *J. Am. Coll. Cardiol.* **75**, 2122–2135 (2020). 32354380 10.1016/j.jacc.2020.02.059 PMC8064770

[10] Das Pradhan, A. et al. *N. Engl. J. Med.* **387**, 1923–1934 (2022). 36342113 10.1056/NEJMoa2210645

